# Effectiveness of Moxibustion Treatment in Quality of Life in Patients with Knee Osteoarthritis: A Randomized, Double-Blinded, Placebo-Controlled Trial

**DOI:** 10.1155/2015/569523

**Published:** 2015-01-21

**Authors:** Xiumei Ren, Chang Yao, Fan Wu, Zhao Li, Jinyun Xing, Haimeng Zhang

**Affiliations:** ^1^Affiliated Hospital of Nantong University, Nantong 226001, China; ^2^Acupuncture-Moxibustion and Tuina College, Shanghai University of Traditional Chinese Medicine, Shanghai 201203, China

## Abstract

*Objective.* To observe the effects of traditional Chinese moxibustion, compared with sham moxibustion, on the quality of life (QOL) in patients with chronic knee osteoarthritis (KOA).* Methods.* This is a randomized double-blinded, placebo-controlled trial. 150 patients with KOA were randomly allocated to either a true moxibustion treatment (*n* = 77) or a sham moxibustion treatment (*n* = 73) three times a week for six weeks. The QOL of patients was evaluated with SF-36 at baseline and 3, 6, and 12 weeks after baseline.* Results.* 136 patients were available for analysis. Participants in the true moxibustion group experienced statistically significantly greater improvement in GH (general health) scores than the sham group at week 6 (*P* = 0.015) and week 12 (*P* = 0.029). Participants in the true moxibustion group experienced statistically significantly greater improvement in VT (vitality) scores than the sham group at week 12 (*P* = 0.042). No significant adverse effects were found during the trial.* Conclusion.* A 6-week moxibustion treatment seems to improve general health and vitality, which are associated with physical and mental quality of life, in patients with KOA up to 12 weeks, relative to credible sham moxibustion. This trial is registered with Clinicaltrials.gov ISRCTN68475405.

## 1. Introduction

The mode of medicine has changed, and so has people's view of their health. The WHO has proposed a new concept of health, which includes not only physical health but also social, mental, and psychological health. While the former method of determining whether certain treatments were effective was plain and had a single focus on physical health, the new method takes multiple influences on patients' social life and mental status into account. Thus, the Social Medical Index of Quality of Life (QOL) has received great attention [1]. In 1993, the WHO defined QOL as “the feeling of an individual about his/her social status, targets, anticipation, standards, and concern in his/her own cultural background and value system. It includes physical health, mental status, degree of independence, social relationship, personal belief and the relativity of their surroundings. It involves a wide range of complex concepts” and this definition is still in use [2].

Knee osteoarthritis (KOA) is commonly seen in middle-aged and elderly people. It causes pain and dysfunction that greatly interfere with patients' quality of life [3, 4]. There is no cure for KOA, and the current methods of treatment are limited to relief of the symptoms. Moxibustion is a common method of treating KOA in East Asia countries, and it is backed by thousands of years of clinical practice. There are many current reports on the clinical efficacy of moxibustion, but reports on the quality of life of KOA patients treated with moxibustion are rarely seen. The current work describes a randomized and strictly controlled clinical trial evaluating the efficacy of moxibustion in the treatment of KOA. The findings on pain and function after moxibustion have been reported in our previously published paper [5]; in this paper, we focus on the evaluation and report of patients' QOLs after moxibustion.

## 2. Methods

### 2.1. Research Design and Setting

This was a double-blinded, placebo-controlled randomized controlled trial (RCT). The study protocol was approved by the Institutional Ethic Review Committee of Chinese Clinical Trials Registry based in Chengdu, China. This RCT was also registered in the Chinese Clinical Trials Registry (ChiCTR-TRC-11001408) on July 6, 2011. Patients were recruited from March 2010 to May 2012 from the Acupuncture and Moxibustion Department of Traditional Chinese Medicine Hospital of Pudong New District, Shanghai; the Community Service Center of Chuansha, Pudong, Shanghai; and the Outpatient Clinic of Acupuncture and Moxibustion of Nantong University's affiliated hospital.

### 2.2. Criteria for Diagnosis, Inclusion, Exclusion, Elimination, Dropping Out, and Termination of Treatment

#### 2.2.1. Diagnostic Criteria

According to the KOA diagnostic criteria issued by the American College of Rheumatology (ACR) in 1986 [6], the following are characteristic of knee osteoarthritis: (1) pain in the knee most days of a month; (2) X-rays showing osteophytes on the sides of the joints; (3) synovial test indicating osteoarthritis; (4) age over 40 years; (5) morning stiffness less than 30 minutes; (6) clicking sound occurring when joint moves. Patients with (1) + (2) or (1) + (3) + (5) + (6) or (1) + (4) + (5) + (6) were diagnosed with knee osteoarthritis.

#### 2.2.2. Inclusion Criteria

Patients were included if they met the diagnostic criteria given above and had (1) knee pain lasting longer than 3 months; (2) moderate or severe knee pain most days of the past month; (3) age under 78 years; (4) willingness to enter a randomized study and to sign the informed consent form.

#### 2.2.3. Exclusion Criteria

Patients were excluded if they (1) had received corticosteroids or intra-articular hyaluronic acid treatment within the past three months; (2) had received joint irrigation or arthroscopy within the past year; (3) had diseases that could interfere the trial and affect the results, such as myocardial infarction, stroke, congestive heart failure, or other severe systemic disease; (4) had been diagnosed with inflammatory arthritis, gout, acute knee damage or other types of arthritis, meniscus, ligament injury, or intrajoint bone fracture; (5) were pregnant; (6) were incapable of filling the scales or who were not willing to be randomized.

#### 2.2.4. Criteria for Rejection, Dropping Out, and Termination

(1) Patients who failed to follow the regimen or who underwent other treatments or drug regimens that could interfere with this trial's final outcome were rejected. (2) Patients who did not have any adverse reaction during the trial but still failed to complete the whole session for other reasons (such as the emergence of other diseases or lost connection) were rejected.

### 2.3. Randomization, Allocation Concealment, and Blinding

*Randomization and Allocation Concealment*. One of the personnel generated random numbers using the RAND function in Excel 2003. The true and sham moxibustion group was assigned with different codes, respectively, and the codes of grouping changed constantly during the RCT. The personnel who recruited the participants were blinded with the group assignment and are only aware of the name and grouping code of each patient. True and sham moxa pillar packs were assigned with the corresponding grouping codes. The practitioners who performed the treatment picked the moxa pillar pack according to the grouping code of the patients when applying treatment.

*Blinding*. During the treatment, the two groups of patients were treated separately by different practitioners. The personnel responsible for the evaluation, recording, and statistical analysis were all blinded to the allocation.

### 2.4. Moxibustion Devices

A commercially available moxibustion device was used (Nanyang Hanyi Moxa Company, Ltd.). It had a cylindrical opening to hold a pillar of moxa and it had an adhesive membrane at the base. During treatment, the device was placed at an acupoint, and the moxa was burned about 8 mm above the skin.

The sham moxibustion device looked exactly the same as the true devices, underwent the same burning process, and produced the same residues, but they had insulated layer in their bases to prevent heat and smoke from coming into contact with the patient's skin. The reliability of this device has been previously tested and validated by Zhao et al. [7].

### 2.5. Treatment Procedure

*(1) True Moxibustion Treatment*. The participant lay supine with the knees slightly flexed with the affected knee(s) exposed. Patients in both groups were treated at three local points, ST 35, EX-LE4, and an Ashi point (the most painful point around knee joint under palpation), in the area of the affected knee(s). Three consecutive moxa pillars were burned at each point. Once the pillar was affixed to a point, the first pillar was lit. When the patient felt burning hot, the pillar was removed and another pillar was affixed and lit. Each treatment session lasted about 20 min. Three pillars were used per acupoint. Treatment was performed three times per week for six weeks. If the patient missed one session he or she was given a session within one week as a makeup.

*(2) Sham Moxibustion Treatment*. The procedure was similar to the true moxibustion group. Because the sham moxibustion device provided insulation from the heat, the patients did not feel hot, and the 3 moxa pillars were allowed to burn out consecutively.

### 2.6. Outcome Measures

SF-36 (Chinese version) was used to assess patients' quality of life (QOL) [8, 9]. This scale covers 8 dimensions (35 items): physical functioning (PF), physical role functioning (RP), bodily pain (BP), general health (GH), vitality (VT), social role functionality (SF), emotional role functionality (RE), and mental health (MH). The 36th item was a self-evaluation of health changes (HT), which was analyzed separately. Each item was scored and the scores were standardized and transformed into final scores on a scale of 0–100, with 0 the worst and 100 the best. The 8 dimensions of SF-36 scale measure physical and mental health. Among these, the dimensions of PF, RP, and BP were mainly found to be associated with physical health, and the dimensions of MH and RE were mainly associated with mental health. The dimensions of SF, VT, and GH were associated with both physical and mental health [10].

The measurements were done before the treatment, at week 3 (midterm), week 6 (end of treatment), and week 12 (6 weeks after the end of treatment). The questionnaire was filled out by the patients with the assistance of the trained practitioners who were blinded with their assignment.

### 2.7. Statistical Analysis

SPSS 17.0 software was used in analysis. Histograms and Q-Q plots were used to assess the distribution of the quality of life outcomes. The outcome data were expressed as mean ± standard deviation (mean ± SD) when the data showed a normal distribution. Otherwise, they were expressed as median (mix, max, and IQR). Chi-square testing was used for analysis of between-group knee conditions and gender distribution. The *t*-test was used for analysis of age and duration of disease. To identify a trend in SF-36 scores, a repeated measure analysis of variance was used for multiple comparisons, and MANOVA was used for between-group assessment. The test level was *α* = 0.05.

## 3. Results

**Figure 1:**
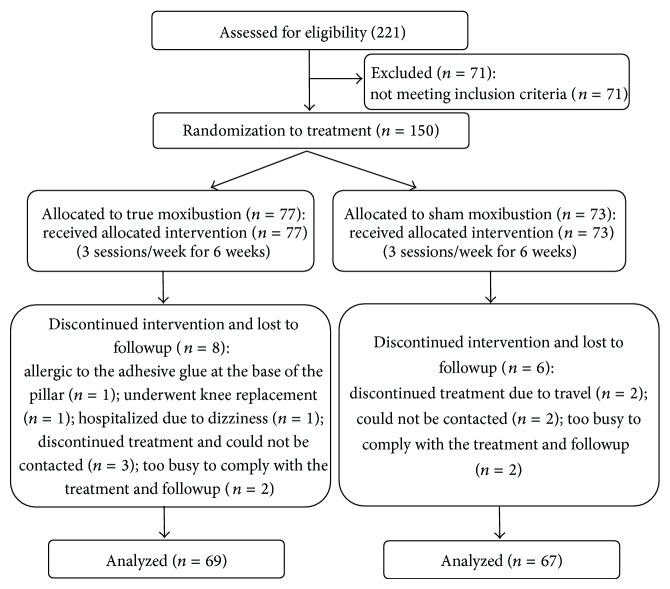
Flow diagram.

A total of 221 potentially eligible patients were screened and enrolled between March 2010 and May 2012. Seventy-one patients were excluded because they did not meet the inclusion criteria. Of the 150 randomly assigned patients, 136 completed the 6-week course of treatment and were assessed at 12 weeks, in which 69 formed the true group and 53 the sham group (Figure 1). The two groups were comparable in demographic characteristics such as gender, age, duration of disease, and the 8 aspects of quality of life at baseline (*P* > 0.05). Baseline characteristics of the patients are presented in Tables 1 and 2.

The sham group's PF at week 6 and BP at both week 6 and week 12 showed statistically significant improvement from baseline (*P* < 0.001, *P* < 0.01). The true moxibustion group's PF, VT, and GH scores at both week 6 and week 12 showed significant improvement from baseline (*P* < 0.01, *P* < 0.05); the true moxibustion group's BP score at all time points (i.e., weeks 3, 6, and 12) showed statistically significant improvement from baseline (*P* < 0.001, *P* < 0.01) (Table 2).

Participants in the true moxibustion group experienced statistically significantly greater improvement in GH (general health) scores than those in the sham moxibustion group at week 6 (*P* = 0.015) and week 12 (*P* = 0.029). Participants in the true moxibustion group experienced statistically significantly greater improvement in VT (vitality) scores than the sham group at week 12 (*P* = 0.042). No significant differences were shown between the two groups for scores in other dimensions (Table 2).

### 3.1. Safety Analysis

In the treatment group, 22 patients experienced blisters of varying sizes from the moxibustion, and 2 patients' blisters were relatively big, but they healed after 1 week. The other 20 patients' blisters all healed within 3 days. All patients considered the blisters nondebilitating and acceptable. No patient experienced discomfort during the moxibustion treatment. Most of the patients regarded moxibustion as a safe procedure and wanted to purchase some moxa pillars and perform moxibustion at home.

### 3.2. Dropouts

The dropout rate was low. By the end of the trial it was 9% (14/150). In the true moxibustion group, 8 of the 77 patients were lost to followup: 1 was allergic to the adhesive glue at the base of the pillar, 1 underwent knee replacement, 1 was hospitalized due to dizziness, 3 discontinued treatment and could not be contacted, and 2 were too busy to comply with the treatment and followup. In the sham moxibustion group, 6 of the 73 patients were lost to followup: 2 discontinued treatment due to travel, 2 could not be contacted, and 2 were too busy to comply with the treatment and followup (Figure 1).

## 4. Discussion

Guidelines for treating KOA regarded relieving pain, maintaining and improving joint's function, and improving patient's life quality as the first priority [11–13]. Our previously published article reported that in this trial, moxibustion reduced pain, improved function, and decreased stiffness for KOA compared with the sham moxibustion procedure [5]. Here, the effect of moxibustion on the quality of life of KOA patients was assessed and reported.

In this double-blind RCT, we found a 6-week course of moxibustion treatment significantly improved vitality and general health, which were both associated with physical and mental quality of life, relative to a believable sham control. Although the effectiveness of moxibustion treatment has been reported in other studies, one recent systematic review showed that the evidence of the larger effect associated with moxibustion is very poor [14]. This was due to small sample size, inadequate use of controls, inadequate followup, and lack of placebo control in previous studies. Reports of quality of life have been very rare for moxibustion treatment [14].

In the present RCT, these limitations were overcome by using rigorous double-blinded, placebo-controlled methods with adequate randomization and a longer followup. The compliance rate of the current RCT was high (91%). In order to facilitate blinding, a validated sham moxibustion device first reported by Zhao et al. in 2006 was used [7]. The appearance, burning process, and residue were the same in the sham and real devices; the only difference was the insulated layer in the base of the sham device. This minimized moxa-produced heat and smoke. The sham control device did produce some warmth, but less than the true moxibustion device. Local skin temperature was measured after each treatment, and active moxibustion produced a temperature of 49.8° on the skin versus the 40.9° produced by the sham treatment [15]. This probably made it difficult for patients to distinguish the true procedure from the sham. However, some physiological effect caused by this lower temperature heat cannot be precluded. To facilitate blinding, only patients who were naïve to moxibustion treatment were included. Communications regarding treatment experiences between the two groups were prevented by treating them on different days. Different groups of practitioners treated the two sets of patients; they had no chance to compare the two devices, saw feedback only from patients of the same group, and were instructed not to discuss the patients' experiences.

The mechanisms of action of moxibustion therapy are still largely unknown. Factors such as temperature, infrared radiation, smoke, odor, and the type of moxa are likely to be involved in the mechanisms by which moxibustion may work [16]. Heat is believed to be the most important factor involved in the effects of moxibustion. Mounting evidence shows that acupuncture relieves pain and improves function in KOA [17–19]. Moxibustion might play a role similar to that of acupuncture stimulation, although its effect on the sensory nerve would be more superficial, due to the heat stimulation on the superficial tissue.

## 5. Conclusion

Moxibustion can be a useful adjunctive treatment for improving physical and mental quality of life in patients with KOA. The findings of the present work should be confirmed and generalized in a larger RCT using a double-blinded, placebo-controlled, multicentered approach.

## Figures and Tables

**Table 1:** Participant demographics and baseline characteristics.

Characteristics	Moxibustion (*n* = 69)	Sham moxibustion (*n* = 67)	*P* value
Age, mean ± SD, y	65.61 ± 7.42	64.06 ± 8.65	0.264
Sex (%)			
Men	20 (29%)	23 (34%)	0.814
Women	49 (71%)	44 (66%)	
Affected knees (%)			
Single knee	55 (80%)	45 (67%)	0.581
Both knees	14 (20%)	22 (33%)	
Duration, mean ± SD, y	6.50 ± 5.86	6.23 ± 7.17	0.121
Quality of life score, mean ± SD, 8 dimensions of SF-36			
PF	57.83 ± 16.88	60.15 ± 17.21	0.428
RP	33.70 ± 37.33	42.91 ± 41.70	0.177
RE	49.76 ± 44.14	56.22 ± 41.52	0.381
VT	53.55 ± 18.19	52.99 ± 18.55	0.858
MH	71.54 ± 14.41	68.78 ± 15.84	0.289
SF	78.62 ± 18.70	75.00 ± 16.71	0.236
BP	58.55 ± 12.82	57.69 ± 18.49	0.751
GH	50.29 ± 15.31	47.99 ± 15.88	0.390

BP: bodily pain; GH: general health; MH: mental health; RE: emotional role functionality; PF: physical functioning; RP: physical role functioning; SD: standard deviation; SF: social role functionality; VT: vitality; y: year.

**Table 2:** Comparison of SF-36 scores (mean ± SD).

8 dimensions of SF-36	Time points	True moxibustion group	Sham moxibustion group	*P* value
PF	Baseline	57.83 ± 16.88	60.15 ± 17.21	0.428
	Week 3	60.65 ± 15.29	61.87 ± 16.87	0.661
	Week 6	63.99 ± 18.24^b^	66.04 ± 14.42^b^	0.467
	Week 12	63.62 ± 17.06^b^	64.10 ± 17.47	0.871

RP	Baseline	33.70 ± 37.33	42.91 ± 41.70	0.177
	Week 3	43.12 ± 41.32	42.16 ± 42.02	0.894
	Week 6	43.48 ± 40.60	45.15 ± 40.88	0.811
	Week 12	41.67 ± 39.22	38.81 ± 40.89	0.678

RE	Baseline	49.76 ± 44.14	56.22 ± 41.52	0.381
	Week 3	59.42 ± 41.96	58.21 ± 44.70	0.871
	Week 6	57.49 ± 43.87	50.25 ± 44.71	0.342
	Week 12	54.11 ± 42.82	48.76 ± 42.37	0.465

VT	Baseline	53.55 ± 18.19	52.99 ± 18.55	0.858
	Week 3	56.16 ± 16.50	56.87 ± 14.40	0.791
	Week 6	59.42 ± 15.87^b^	56.49 ± 14.62	0.265
	Week 12	61.30 ± 16.22^b^	55.52 ± 16.68	0.042^d^

MH	Baseline	71.54 ± 14.41	68.78 ± 15.84	0.289
	Week 3	69.33 ± 13.35	66.27 ± 15.26	0.214
	Week 6	68.81 ± 12.75	66.99 ± 14.43	0.435
	Week 12	68.81 ± 13.95	66.33 ± 17.80	0.366

SF	Baseline	78.62 ± 18.70	75.00 ± 16.71	0.236
	Week 3	78.26 ± 15.99	76.49 ± 17.21	0.536
	Week 6	79.17 ± 13.33	78.54 ± 15.05	0.799
	Week 12	76.45 ± 14.93	75.00 ± 17.13	0.600

BP	Baseline	58.55 ± 12.82	57.69 ± 18.49	0.751
	Week 3	63.62 ± 12.46^b^	61.72 ± 15.72	0.434
	Week 6	67.54 ± 14.45^a^	65.93 ± 13.53^a^	0.506
	Week 12	66.56 ± 14.68^a^	65.60 ± 16.83^b^	0.723

GH	Baseline	50.29 ± 15.31	47.99 ± 15.88	0.390
	Week 3	49.20 ± 15.35	46.87 ± 14.30	0.360
	Week 6	54.42 ± 15.92^c^	48.43 ± 12.10	0.015^d^
	Week 12	54.57 ± 14.67^c^	48.88 ± 15.44	0.029^d^

BP: bodily pain; GH: general health; MH: mental health; RE: emotional role functionality; PF: physical functioning; RP: physical role functionality; SF: social role functionality; VT: vitality.

^a^Compared with pretreatment: *P* < 0.001; ^b^compared with pretreatment: *P* < 0.01; ^c^compared with pretreatment: *P* < 0.05; ^d^b-group comparison: *P* < 0.05.
